# How mood shapes belief updating bias in depression

**DOI:** 10.3758/s13415-025-01297-x

**Published:** 2025-04-30

**Authors:** Hugo Bottemanne, Solène Frileux, Caroline Sevoz-Couche, Yann Pelloux, Romain Colle, Emmanuelle Corruble

**Affiliations:** 1 Service Hospitalo-Universitaire de Psychiatrie de Bicêtre, Mood Center Paris Saclay, Assistance Publique-Hôpitaux de Paris, Hôpitaux Universitaires Paris-Saclay, Hôpital de Bicêtre, 94275 Le Kremlin Bicêtre, France; 2 MOODS Team, INSERM 1018, CESP (Centre de Recherche en Epidémiologie Et Santé Des Populations), Faculté de Médecine Paris-Saclay, Université Paris-Saclay, 94275 Le Kremlin Bicêtre, France; 3 Service Hospitalo-Universitaire de Psychiatrie de Versailles, Centre Hospitalier de Versailles, Le Chesnay, France

**Keywords:** Depression, Belief, Belief updating, Belief formation, Major depressive disorder

## Abstract

Major depressive disorder (MDD) is characterized by mood-congruent beliefs, such as devaluation, unworthiness, helplessness, pessimism, or guilt. These depressive beliefs could cause and maintain emotional and behavioral disturbances, playing a central role in MDD prognosis. Drawing on studies exploring how mood affects information processing, we propose a mechanistic theory of belief updating in depression. First, we show how depressive beliefs are formed in environments where negative stimuli are weighted more heavily. Second, we demonstrate how depressed individuals often hold rigid negative metacognitive priors that inhibit belief updating. Third, we clarify how negative beliefs can be generated internally through repetitive, self-focused cognitive patterns. Finally, we critically examine the limitations of current experimental paradigms used to assess belief updating, highlighting methodological constraints and potential confounds. Based on these insights, we outline future research directions to refine experimental designs and improve our understanding of mood-congruent belief updating in depression.

## Introduction

Major depressive disorder (MDD) is a debilitating condition that profoundly impairs psychosocial functioning and quality of life (Friedrich, 2017). It is marked by emotional, cognitive, and behavioral disturbances, including persistent sadness, rumination, and social withdrawal (Malhi & Mann, 2018). A defining feature of MDD is the presence of maladaptive depressive beliefs, such as self-devaluation, unworthiness, helplessness, pessimism, and guilt (Neelapaijit et al., 2017; Orchard & Reynolds, 2018). These beliefs may contribute to the onset and persistence of emotional and behavioral dysfunctions, increasing the risk of suicide attempts (da Silva et al., 2018; LeMoult & Gotlib, 2019) and playing a pivotal role in the prognosis (Chahar Mahali et al., 2020) and overall morbidity of MDD (Thimm et al., 2013). Despite their clinical significance, the mechanisms linking mood and belief formation remain largely unexplored (Teasdale, 1983).

In the field of psychology, beliefs are regarded as representational constructs—mental states that encode propositional content about the world, serving as cognitive frameworks through which individuals interpret and interact with their surroundings (Yon et al., 2020). Depressive beliefs, specifically, are often described as mood-congruent, meaning that their representational content aligns with and reflects the individual’s prevailing negative emotional state (Kube et al., 2020). Shaped by sociocultural contexts, these negative beliefs often reinforce pessimistic interpretations of reality, distort reasoning processes and perpetuate depressive states through feedback loops of negative perception and inference (Beck, 1987; Evans et al., 2005).

Understanding the origins of depressive beliefs is essential for insight into how these maladaptive representations emerge and persist. Growing research on belief updating explores how individuals revise their beliefs in response to new information (Scheffer et al., 2022; Sharot et al., 2022). Findings indicate that individuals with MDD are less likely to update negative beliefs when confronted with positive information (Kube & Rozenkrantz, 2020). This evolving field is crucial for understanding MDD, because it reveals how impairments in belief updating contribute to biased perceptions of reality (Kube et al., 2017). However, a comprehensive model explaining the origins of these mechanisms is still lacking.

Drawing on studies exploring how mood affects information processing, we propose a mechanistic theory of belief updating in MDD. We first describe the characteristics of depressive beliefs and a cognitive model of belief formation. We show how the valence of prediction errors, the hierarchy of belief, and cognitive feedback lock may produce affective-biases in belief-updating. Finally, we critically examine the limitations of current experimental paradigms used to assess belief updating, highlighting methodological constraints and potential confounds. Based on these insights, we outline future research directions to refine experimental designs and improve our understanding of mood-congruent belief updating in depression.

## Negative beliefs in depression

Ranges of classical theory about depressive belief emerge from psychiatrist Aaron Beck’s original theory (Beevers, 2005), distinguishing two main constructs: core-beliefs (or schemas) and automatic-thoughts (Beck, 1987). Beck defines core-beliefs as rigid, negative attitudes about the self and its relationship to the world, shaped during childhood and early adulthood, and remaining relatively stable throughout life (Beck, 1963). He suggests that these beliefs bias perception and cognition, leading to unrealistic and negatively distorted interpretations that emphasize the threatening, aversive, or uncertain aspects of the environment (Beck, 1976), driving mood-congruent automatic thoughts when activated by life experiences (Neelapaijit et al., 2017).

Aaron Beck initially proposed distinguishing a triad of content for depressive beliefs (Beck, 1976): oneself (depreciation, worthlessness, helplessness, unworthiness); world (rejection, wickedness, aimless, useless world); and future (aversive events, powerlessness, unpredictability). However, distinguishing belief themes is challenging, because they often overlap. Most belief themes are indirectly connected to the self, the world, and the future (Philippi et al., 2018; Sarsam et al., 2013). Furthermore, many depressive beliefs are characterized by a sense of losing control, both over the environment and one’s actions. This perception diminishes the ability to act effectively in the world and strengthens the certainty of being unable to cope with stressful situations (Andersen & Lyon, 1987; Bottemanne et al., 2022a; Stephan et al., 2016).

A number of research have focused on negative beliefs about the future (Andersen & Lyon, 1987; Thimm et al., 2013). Studies have shown that depressed patients perceive future negative events as more likely than positive ones (Alloy & Ahrens, 1987; Pyszczynski et al., 1987; Thimm et al., 2013), anticipate negative events more frequently (Herwig et al., 2010), and believe positive events are less likely to happen to them compared with others (Alloy & Ahrens, 1987; Pyszczynski et al., 1987). These quantitative biases are accompanied by qualitative distortions, with depressed individuals anticipating more intense negative emotions in response to future events (Wenze et al., 2012), expecting these emotions to be stronger than those experienced by the general population (Miranda et al., 2008), and perceiving future positive events as less positive in nature (MacLeod & Salaminiou, 2001).

These negative beliefs have been correlated with the severity of MDD and the onset of suicide (Andersen et al., 1992; Horwitz et al., 2017; Strunk et al., 2006). They may influence negatively the perception of current and past experiences and thereby induced behavioral attitudes, such as withdrawal and isolation, that further reinforce negative beliefs (Kube et al., 2020). In the following section, we will delve deeper into the mechanisms that drive the formation of negative beliefs, shedding light on the processes that underpin their emergence and persistence.

### Cognitive processes of belief formation

Since Beck’s theory, cognitive framework of belief formation has advanced to explore how beliefs are constructed, maintained, and distorted over time (Bottemanne, 2024; Porot & Mandelbaum, 2021). This framework adopts an implicit (unconscious, subpersonal), probabilistic concept of beliefs, distinct from the traditional notion of belief in psychology (Yon et al., 2020). Beliefs are understood in terms of probability densities over events (observations or states), modeled as prior predictions that are integrated with incoming sensory information to generate the brain’s most plausible explanation for its experiences, and encoded in the brain as activity patterns in neural networks (Friston, 2005). Additionally, this framework posits that psychological beliefs (explicit propositional representations) emerge from probabilistic beliefs (implicit probability distributions), analogous to how dispersed sensory signals are integrated into a cohesive perceptual representation (Bottemanne, 2024).

Most contemporary theories of belief are grounded in predictive processing (PP), which proposes that the brain uses beliefs to interpret incoming sensory information, continuously refining these beliefs by integrating external evidence (Kube et al., 2020). Probabilistic beliefs are modeled as top-down predictions processed by higher cortical areas to predict bottom-up sensory inputs, producing prediction errors (Yon et al., 2020), according to a hierarchical anatomical gradient in a constant feedforward/feedback loop (Mumford, 1992). It is hypothesized, supported by experimental evidence, that hierarchical inference is associated with the excitation-inhibition balance maintained by the synaptic activity of columns of pyramidal (glutamatergic) neurons and inhibitory (GABAergic) interneurons distributed throughout the superficial and deep layers of the cortex (Bastos et al., 2012; Sah et al., 1989).

These inferences depend on precision, with the brain adjusting the weight of prediction errors according to their confidence, balancing prior beliefs with new evidence (Kanai et al., 2015). Precision weighting depends on interactions between inhibitory interneurons and slower modulatory neurotransmitters, such as dopamine, serotonin, noradrenaline, or acetylcholine (Iglesias et al., 2021; Redgrave & Gurney, 2006; Schwartenbeck et al., 2015). This mechanism is crucial in uncertain environments, with high-precision errors or low-precision predictions favoring updates, and the reverse reducing them. Precision control optimizes the balance of predictions and errors in the model, ensuring proper inference by calibrating precision to match the signal-to-noise ratio of sensory inputs (Friston & Kiebel, 2009). This mechanism prevents the incorporation of irrelevant or noisy components during information processing.

**Fig. 1:**
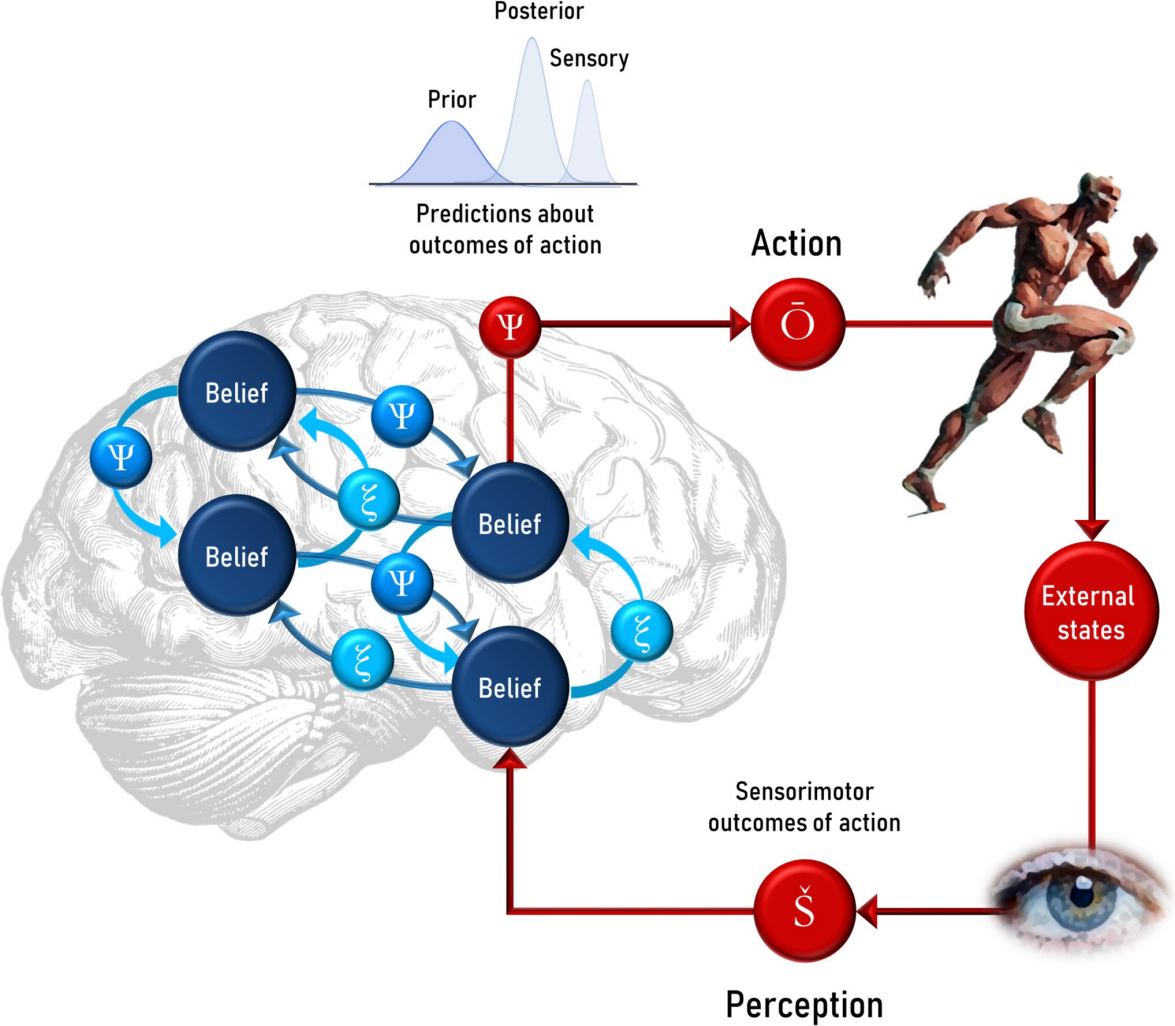
Hierarchical belief updating in predictive processing framework. Most cognitive theories of belief formation propose that the brain encodes a hierarchy of beliefs to predict incoming sensory information and guide goal-directed actions. Top-down predictions (Ѱ) are compared to sensory signals (Š) resulting from actions (Ō), with discrepancies generating bottom-up prediction errors (ξ) that travel through the cortical hierarchy to update beliefs. This process depends on the precision of predictions, where more precise predictions are harder to update, while lower precision facilitates belief revision. This neurophysiological model of belief updating is often described through Bayesian inference, where the posterior belief emerges from combining prior beliefs (predictions) and sensory evidence, with hierarchical inference updating beliefs to find the best balance between the two. The anatomical location in the brain is schematic and not based on neuroanatomical correlates of these processes. Š: Sensory data; Ѱ: Prediction; ξ: Prediction error; Ō: Action

Through this theory, a neurophysiological model for belief generating and updating in the brain is conceptualized and sometimes modeled as a Bayesian inference (Friston, 2005) (see Fig. 1 for a detailed illustration).

The PP framework provides a novel lens for understanding depression, yet it is important to contextualize this approach alongside competing theories, such as those emphasizing deficits in reward processing (Admon & Pizzagalli, 2015; Whitton et al., 2015). Reward-based theories propose that depression is characterized by reduced sensitivity to positive outcomes, impairments in reinforcement learning, and a diminished ability to anticipate or respond to rewarding stimuli (Admon & Pizzagalli, 2015). These models are supported by evidence of altered activity in dopaminergic pathways and reduced neural responses in reward-related regions, such as the ventral striatum (Ubl et al., 2015). While these theories offer a robust explanation for certain aspects of depression, the PP framework extends beyond them by addressing how both positive and negative information is integrated into maladaptive priors—rigid and overly negative expectations—which influence not only the processing of reward but also the amplification of aversive stimuli (Clark et al., 2018; Hesp et al., 2020). This dual effect suggests that depression involves a fundamental imbalance in hierarchical inference, rather than a unidirectional deficit in reward processing. For example, whereas reward theories might predict reduced responses to positive prediction errors, the PP framework predicts an overarching bias in precision-weighting that impacts the processing of both positive and negative prediction errors (Clark et al., 2018; Harmer & Browning, 2020).

This distinction allows the PP framework to generate broader predictions that complement and enhance existing models. In the following sections, we will build on this cognitive theory framework of belief formation to explore the development of negative beliefs in depression.

### Affective valence bias

As theorized by Aaron Beck, research has shown that individuals with depression tend to verify their self-representations by seeking negative—rather than positive—appraisals, and they often prefer unfavorable evaluations and relationships with those who offer such assessments (Swann et al., n.d.) Their desire to confirm negative self-related judgments overrides the need for praise, and this drive to obtain confirmation intensifies when others’ positive evaluations challenge their self-view. Additionally, they exhibit a decrease in reward-related associative learning and an increase in loss-related associative learning (Ubl et al., 2015). Taken together, these findings suggest that individuals with depression struggle to successfully integrate new information, meaning they cannot adjust their negative self-representations to align with new, contradictory information. This phenomenon reflects a hypersensitivity to negative outcomes in depression, linked to the association between increased avoidance learning and larger error signal amplitudes (Cavanagh et al., 2011).

Within the cognitive framework of belief formation, maladaptive depressive beliefs can be understood as arising from distorted inferences and precision-weighting (Clark et al., 2018). These processes have been explored through belief updating tasks (Hobbs et al., 2022; Scheffer et al., 2022; Sharot et al., 2022). These tasks consist of an initial belief assessment, the presentation of new information (feedback), and a subsequent reassessment of beliefs after integrating this information, with updates influenced by its valence (positive or negative) (Bottemanne et al., 2022b; Kube & Rozenkrantz, 2020). Most of the experimental tasks deal with three broad belief themes: future life events (BUT task) (Bottemanne et al., 2022b; Miranda et al., 2017; Sharot et al., 2011a), one’s own performances (EXPECD task) (Feldmann et al., 2022; Groth & Rief, 2022b; Kube et al., 2018) and interpersonal situations (BADE task) (D’Astolfo et al., 2020; Deng et al., 2022; Everaert et al., 2018; Groth & Rief, 2022a; Kube et al., 2022; Liknaitzky et al., 2017).

The most widely used experimental task is the BUT (Belief Updating Task). It assesses belief updating about prospective beliefs, examining how individuals adjust their estimate about the probability of future event when new (positive or negative) information is presented. In the general population, there is a valence bias in belief updating characterized by an increased sensitivity to a positive feedback and decreased sensitivity to a negative (Kuzmanovic et al., 2018; Sharot et al., 2011b). This updating bias is assumed to be the source of positively biased belief in healthy subjects for a range of themes, such as health status (Taylor et al., 2000), love relationships (Baker & Emery, 1993), or professional success (Puri & Robinson, 2007). Studies have shown that the bias disappear in MDD (Garrett et al., 2014; Korn et al., 2014; Sharot et al., 2011b). Compared with control subjects, depressed patients update their negative beliefs less after receiving positive feedback, and this decrease is positively correlated with the clinical severity of depressive symptoms (Garrett et al., 2014; Korn et al., 2014).

Another frequently used task is the EXPECD task (Experimental Paradigm to investigate Expectation Change in Depression), designed to mathematically model belief updating in a performance-related but nonself-referential context (Feldmann et al., 2022). It evaluates belief updating regarding performance expectations based on (false) feedback, such as on an emotional intelligence test or emotion recognition task (Kube et al., 2018). Across a number of trials, participants estimated their expected performance on a test by providing a prior belief distribution—specifically, the most likely number of correct answers (prior mode) and their confidence in this estimate (precision), and they received fabricated performance feedback. Finally, they updated their performance beliefs for the next trial to assess how prior beliefs were revised in response to feedback (posterior beliefs). Studies found that healthy participants adjusted their performance expectations according to the positive feedback, whereas patients maintained their initial belief (Kube et al., 2019b). Another study identified a correlation between the severity of depressive symptoms and reduced updating of negative belief (Kube et al., 2021). With the same paradigm, another study found no difference between MDD patients and healthy controls in updating beliefs following negative feedback (Kube et al., 2019a).

Finally, a third frequently used task is the BADE task (Bias Against Disconfirmatory Evidence), which assesses belief updating in interpersonal situations. It examines how individuals adjust their interpretations of an ambiguous interpersonal scenario when new evidence is presented. (Everaert et al., 2018). The BADE task is particularly useful in exploring how cognitive biases, such as the tendency to disregard evidence that contradicts existing beliefs, affect the interpretation of social situations. Nonclinical studies have shown that individuals with depressive symptoms have difficulty updating negative interpretations when presented with contradictory positive information, a phenomenon known as cognitive immunization (Rief et al., 2015). However, depressive symptoms do not appear to impair the revision of positive interpretations in response to new negative information (Deng et al., 2022; Everaert et al., 2018, 2020). Moreover, the negative bias could predict the onset of suicidal ideation in clinical population (Everaert et al., 2021).

Overall, these results show diverse effects. Some studies suggest that belief updating in MDD is not driven by heightened sensitivity to negative feedback but rather by a reduced capacity to revise negative beliefs in response to positive feedback (Hobbs et al., 2022; Korn et al., 2014), whereas other studies suggest greater updating following negative information (Bottemanne et al., 2022b). This valence-dependent asymmetry may result in an undervaluation of positive stimuli and an overvaluation of negative stimuli, with idiosyncratic variations of this bias across individuals, ultimately reinforcing maladaptive negative beliefs. (Sharot & Garrett, 2016). These behavioral findings are further supported by neuroimaging results, which reveal the neural mechanisms underlying belief updating in depression. Specifically, one study found strong neural coding of prediction errors in response to both positive information (left inferior frontal gyrus and bilateral superior frontal gyrus) and negative information (right inferior parietal lobule and right inferior frontal gyrus) about the future (Garrett et al., 2014).

Moreover, recent advancements in neuroimaging have shed light on the brain regions involved in encoding valence (Sharot & Garrett, 2016). Studies indicate that the ventromedial prefrontal cortex (vmPFC) plays a key role in representing the expected value of stimuli (Kuzmanovic et al., 2018), whereas the ventral striatum, which is involved in reward processing, tracks prediction errors related to rewards and punishments, prompting the brain to adjust its expectations accordingly (Zhu et al., 2012). Another study found that the anterior insula and the amygdala, known for its role in threat detection, processes negative prediction errors, signaling the brain to revise expectations when faced with potential danger or loss (Müller-Pinzler et al., 2022). Together, these brain regions form a network essential for the valuation of signals and the updating of predictions based on ongoing environmental feedback (Cooney et al., 2010).

In depression, disruption of activity in these regions could lead to an overweighted negative valence feedback, resulting in an overreliance on prior negative beliefs and difficulties in updating them in response to positive feedback (Kube, 2023). The dysfunction of ventromedial prefrontal cortex may reduce the distinction between positive from negative valence in MDD (Kuzmanovic et al., 2018), falsely assigning negative valence to neutral signals, whereas an increase in amygdala activity in response to negative stimuli could reinforce the emotional consequences of these negative signals (Siegle et al., 2002). Moreover, anterior cingulate cortex is involved in conflict monitoring and error detection, which may contribute to a heightened sensitivity to negative signals while reducing the sensitivity to positive ones (Cooney et al., 2010). This impaired prediction-error signaling is thought to contribute to the persistence of negative biases and maladaptive beliefs in depression.

### Belief network distortion

In addition to the differential treatment of information based on valence, other mechanisms rooted in the hierarchical organization of belief networks may play a role. Rather than merely favoring positive over negative updates, the brain’s belief system operates within a structured framework, where certain priors exert stronger constraints on belief revision than others. This suggests that biased belief updating may emerge not only from valence-driven filtering but also from the hierarchical interplay between higher-order expectations and incoming evidence (Huntley & Fisher, 2016; Kube et al., 2017).

Experiments have revealed that the connections between beliefs form a filtering system that selectively influences how new information is incorporated (Kube et al., 2020). Beliefs that are logically interconnected tend to resist information that contradicts them, while they more readily accept information that aligns with their existing framework (Kube et al., 2019c). This process can lead to a reinforcement of prior beliefs, especially when individuals are confronted with evidence that challenges their worldview. In such cases, instead of reconsidering their views, individuals may strengthen them, resulting in a feedback loop known as the “worldview backfire effect” (Cook & Lewandowsky, 2016; Lewandowsky et al., 2012, 2018; Nyhan & Reifler, 2010). This selective updating mechanism leads to a biased integration of information, where the emotional valence of the new information plays a pivotal role in determining whether it is accepted or ignored, further shaping the individual's belief system in a way that favors consistency over accuracy.

**Fig. 2:**
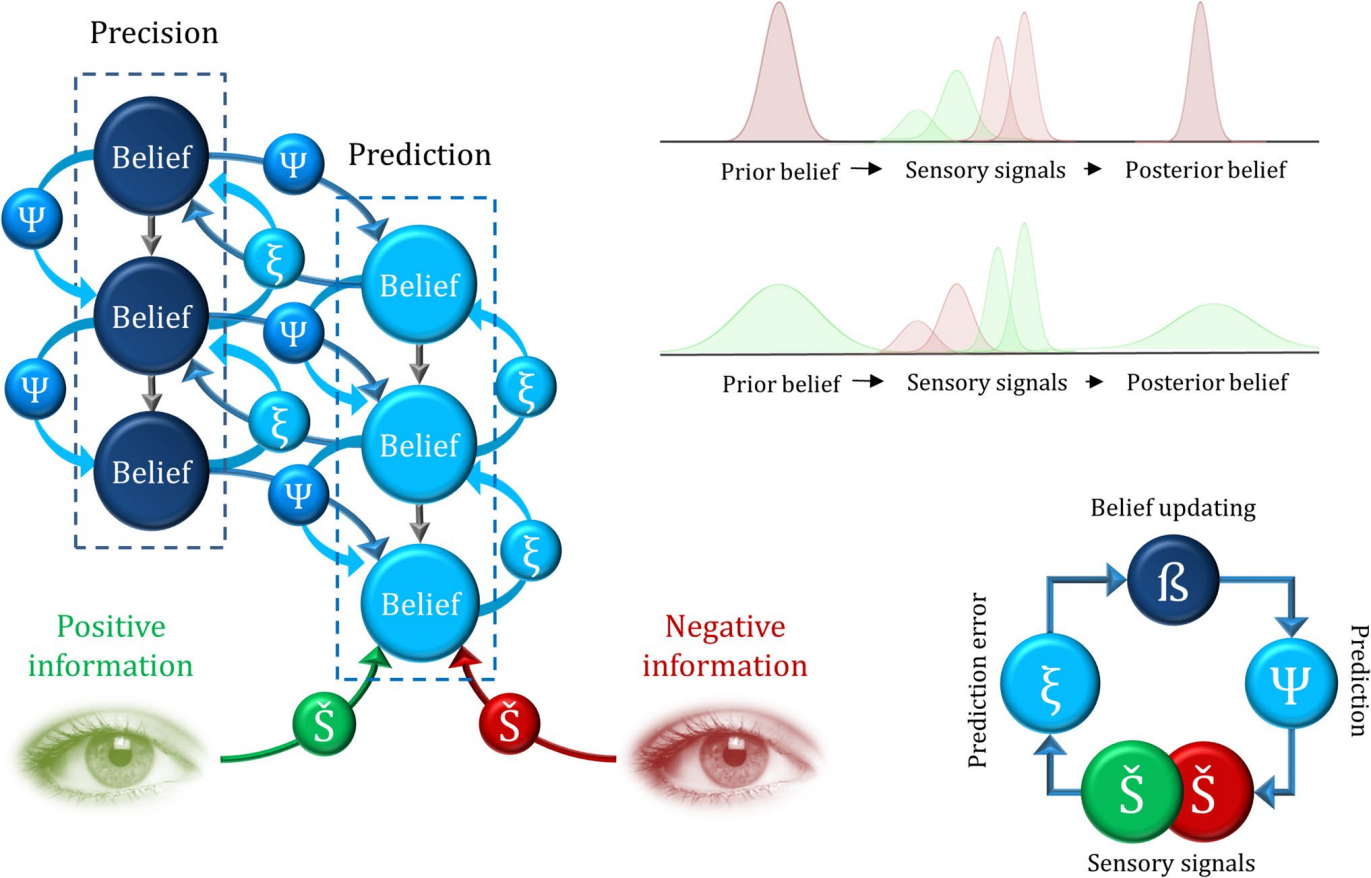
Role of precision in valence-biased belief updating. This schematic illustrates how belief formation is influenced by the valence of information. On the left side, the belief network is depicted with two key components: the first column represents the precision of prior beliefs, which modulates the rate of belief updating, while the second column represents predictions about sensory signals. On the upper right, Bayesian probability distributions are shown as a function of valence: high-precision distributions are narrower and more peaked, whereas low-precision distributions are broader and flatter. The model posits that high precision in negative beliefs facilitates the integration of negative information, while low precision in positive beliefs constrains the incorporation of positive information. Together, these mechanisms contribute to a valence-biased belief updating process. Finally, on the lower right, the belief updating loop is illustrated: predictions are generated based on priors, and discrepancies between these predictions and incoming sensory information produce prediction errors, which in turn drive the revision of prior beliefs. Š: Sensory data; Ѱ: Prediction; ξ: Prediction error; ß: Belief updating

This distortion, related to the belief network, can be modeled using the concept of precision-weighting (Friston, 2012). Within this framework, inferences made within the belief hierarchy are weighted by metabeliefs that encode precision. These metabeliefs reflect the confidence or certainty in the information, influencing how strongly certain beliefs are updated. When precision-weighting is applied, more certain beliefs—often those aligned with an individual’s affective state or prior experiences—receive greater influence in the belief updating process, reinforcing biases and shaping the overall belief structure (Hesp et al., 2020). This precision-weighting bias may produce an epistemic resistance to contradictory information (Iijima et al., 2017), reinterpreting counterevidence by treating positive information as an anomaly rather than a general rule. Moreover, it may explain why depressed patients’ degree of belief for negative content is higher than nondepressed individuals’ (Andersen & Lyon, 1987) (see Fig. 2 for a detailed illustration).

At the computational level, this predictive processing theory of belief formation suggests that beliefs are shaped in response to abnormal prediction-error signals through a three-stage dynamic process: the accumulation of prediction-error signals alerts the system that something needs to be clarified or learned, disrupting the processes involved in automatic and habitual learning. This uncertainty can be exacerbated by issues in the salience system (Shao et al., 2018), with attention being directed toward aberrant stimuli, increasing prediction errors and prompting a need to make sense of the situation. Ultimately, this leads to the generation of depressive beliefs aimed at resolving overwhelming uncertainty and the unpredictability of both the external and internal world, reinforcing previously acquired beliefs whenever previously inexplicable events are encountered.

It has been suggested that the aberrant integration of dendritic inputs in supragranular pyramidal cells, or pre- and postsynaptic deficits in neural pathways, may disrupt the hierarchical inference process based on predictions and prediction errors (Haker et al., 2016). An alternative hypothesis proposes that the precision of predictions and prediction errors could be abnormally tuned within the predictive hierarchy, potentially due to dysregulation of monoaminergic neurotransmitters (Clark et al., 2018). This could imply that negative predictions are overly precise, while the precision of prediction errors is diminished, leading to perceptual and attentional biases toward negative stimuli (De Raedt & Koster, 2010) and a systematic negative bias in belief updating (Chekroud, 2015). The disruption in precision-weighting might particularly affect dopaminergic signaling in regions associated with reward processing, such as the nucleus accumbens and the caudate nucleus (Pizzagalli et al., 2009). In MDD, reward circuitry is compromised, with hypersensitivity to punishments and hyposensitivity to positive rewards, impairing effective reward learning (Whitton et al., 2015). This involves inhibition of PE processing in the reward system within the ventral striatum, mediated by the prefrontal cortex (Kube et al., 2020). Collectively, disruptions in the activity of these areas in MDD could hinder the brain's ability to suppress negative information.

### Cognitive feedback lock

Other factors contributing to the impaired belief updating observed in MDD include the disruption of neurocognitive processes, particularly in memory, attention, and executive functions, alongside the affective valence bias and dysfunctional metabeliefs (Hammar et al., 2022). Memory disturbances are common, with a tendency to preferentially recall negative information while struggling to suppress it, leading to a biased reconstruction of past experiences (Joormann & Gotlib, 2008; Mathews & MacLeod, 2005). Attention is also affected, as individuals tend to focus disproportionately on negative or threatening stimuli, ignoring or failing to process more neutral or positive information (Keller et al., 2019). Additionally, executive functions, such as cognitive flexibility and the ability to update beliefs, are impaired, making it harder to disengage from negative information and adapt to new experiences (Koster et al., 2011a). These neurocognitive deficits disrupt the normal processing of information, exacerbating the negative biases that underlie the persistent, maladaptive beliefs characteristic of depression.

In addition to these neurocognitive disturbances, rumination, characterized by perseverative, self-referential, and negatively biased information processing, is a hallmark feature of MDD, playing a crucial role in its onset, persistence, and recurrence (Kircanski et al., 2012; Nolen-Hoeksema et al., 2008). During rumination, individuals compulsively revisit autobiographical, negatively charged material related to past and present struggles (Alderman et al., 2015; Davidson et al., 2002). This perseverance is reinforced through affective and cognitive feedback loops, ultimately maintaining and exacerbating negative content. As a result, individuals struggle to disengage from a negative, self-referential focus (Koster et al., 2011b; Ruscio et al., 2011). The relationship between rumination and MDD appears bidirectional (Whisman et al., 2020), with rumination serving as a significant risk factor for MDD (Stange et al., 2016) and acting as a key mediator between neuroticism and depressive symptoms (Lu et al., 2017).

A study shows that ruminative processes restrain the use of external sensory inputs, thus increasing the weight of subjective information in belief-updating process (Marroquín & Nolen-Hoeksema, 2015). Also, depressed patients who manage to disengage from their ruminations show less negative beliefs than patients harboring an exclusive self-referenced treatment (Pyszczynski et al., 1987). These results suggest that self-focused rumination disturbs the information processes involved in belief updating, increasing the strength of depressive beliefs (Marroquín et al., 2016). Supporting this hypothesis, a study conducted in a naturalistic experimental setting found distinct mechanisms underlying pessimistic beliefs in anxiety and depression. Specifically, anxiety was linked to negatively biased belief updating, where individuals more readily incorporated negative information into their beliefs than positive information. In contrast, depression was associated with pessimistic prior beliefs—preexisting assumptions formed before receiving explicit feedback, likely shaped by past experiences or ingrained cognitive schemas (Gagne et al., 2022).

Metacognitive beliefs about the usefulness of rumination are crucial in sustaining dysfunctional information processing in depression. Depressed individuals often view rumination as a helpful coping strategy or a way to resolve problems (Nolen-Hoeksema et al., 2008), which reinforces the rumination loop (Yilmaz et al., 2015). Paradoxically, they also hold contradictory metabeliefs: negative views, such as seeing rumination as a sign of weakness, and positive ones, such as believing it leads to solutions, both of which reinforce the cycle (Aslan & Baldwin, 2021; Papageorgiou & Wells, 2003). Moreover, the inability to revise these metabeliefs is linked to the intensity of depressive symptoms (Takano et al., 2019). Disruptions in updating metacognitive beliefs in MDD create a self-reinforcing cycle between rumination and negative beliefs, despite evidence that rumination does not resolve problems (Matsumoto & Mochizuki, 2018).

### Limitations of experimental paradigms

The experimental literature on belief updating often relies on tasks designed to probe probabilistic inference in highly controlled settings (Bottemanne et al., 2022b; Korn et al., 2014; Kube et al., 2019c). While these tasks provide valuable insights into the cognitive processes underlying belief updating, they face significant limitations. Specifically, many paradigms oversimplify the complex, iterative nature of belief formation and revision in real-world contexts.

Belief updating tasks typically present participants with discrete, static scenarios requiring explicit judgments about confidence or probability, treating beliefs as distinct, measurable variables (Garrett et al., 2014). Although this approach streamlines experimental design, it neglects the fact that beliefs often lie on a continuum of implicitness and fails to fully capture the continuous and dynamic nature of belief updating, where beliefs are adjusted in response to fluctuating, often ambiguous sensory and contextual informations (Bastos et al., 2012). Additionally, belief updating tasks often rely on self-report measures, such as confidence ratings or stated probabilities (Bottemanne et al., 2022b; Korn et al., 2014). While these measures offer straightforward insight into explicit belief states, they are limited in capturing implicit probabilistic beliefs (Bastos et al., 2012). Beliefs are shaped by unconscious priors, contextual cues, and affective states, which are difficult to quantify using traditional methods. Implicit inferences, which operate beneath conscious awareness, are central to hierarchical Bayesian models but are largely inaccessible to self-report techniques (Friston, 2005).

This dependence on explicit measures raises several concerns. First, it may oversimplify key dynamics, such as the interaction between implicit priors and explicit posteriors, or the gradual accumulation of evidence over time (Müller-Pinzler et al., 2022). Second, self-reports are prone to introspective inaccuracies and biases, including social desirability or demand characteristics (Sharot & Garrett, 2022). Third, they may fail to capture the underlying distributions of prior beliefs and prediction errors that drive belief updating (Bottemanne et al., 2022b). These limitations suggest that self-report measures provide only a partial—and potentially distorted—perspective of the belief updating process. A more sophisticated approach would involve integrating methods capable of inferring implicit belief states through behavioral modeling, complementing traditional self-report measures (Adams et al., 2016).

Unlike tasks with simple stimuli, belief updating tasks that incorporate explicit probabilities and realistic life events (e.g., being diagnosed with cancer) engage socially significant situations, which is especially pertinent in the context of depression. However, a key limitation is that the variability in the content of these events may introduce biases in the evaluation of updating mechanisms. By utilizing ecologically relevant events, controlling the intensity of negativity for each belief and associated information becomes challenging. Moreover, idiosyncratic factors—shaped by participants'individual life experiences—can influence the valence of prior beliefs, and MDD is often linked to disturbances in the perceived valence and desirability of certain life events (Nilsson et al., 2023). To reduce this classification bias, one possible solution is to have participants indicate whether each event was perceived as positive or negative at the conclusion of the task and rate the intensity of the valence on a Likert scale.

Addressing these limitations requires the development of more sophisticated and ecologically valid belief updating tasks. For example, future research could incorporate paradigms that simulate dynamic, real-world contexts where beliefs are updated continuously and hierarchically. In addition, combining self-report data with implicit measures—such as reaction times, eye-tracking, or neural correlates of belief updating—could provide a more comprehensive understanding of the underlying processes.

### Testing the boundaries of belief updating bias

Beck’s cognitive model of depression has long emphasized the role of maladaptive schemas and cognitive biases in shaping individuals’ negative interpretations of themselves, their environment, and the future (Beck, 1987). While Beck’s framework accounts for the role of cognitive distortions in depression, cognitive theories of belief formation extend these ideas by emphasizing precision-weighting, which refers to the brain’s ability to modulate the relative influence of prior beliefs (top-down signals) and sensory evidence (bottom-up signals) during belief updating (Friston et al., 2017). In healthy cognition, precision-weighting enables individuals to dynamically adapt their beliefs based on the reliability of incoming sensory information and the context in which it is encountered (Sharot et al., 2022).

From this perspective, cognitive biases, such as attentional and interpretational biases, can be seen as the result of an imbalance between prior expectations and incoming evidence, favoring the former even when it conflicts with reality (Harmer & Browning, 2020). Negative beliefs in MDD can be understood as priors with overly high precision, restricting the influence of new sensory information (Clark et al., 2018). Our central hypothesis posits that depressive biases specifically emerge from dysfunctional interactions between high-level meta-beliefs and low-level perceptual updates. This hierarchical framework closely aligns with Beck’s concept of rigid negative thinking, while offering additional explanatory depth by quantifying the interaction between expectations and evidence (Kube et al., 2020).

Advances in computational modeling provide tools to simulate and quantify these processes by using belief updating tasks in which the reliability of sensory inputs is systematically manipulated, alongside dynamic causal modeling and neuroimaging (Adams et al., 2016). It is hypothesized that depressed individuals will place more weight on negative information in the formation of their beliefs, leading to a stronger bias towards negativity (Feldmann et al., 2022). Depressed participants are expected to exhibit a reduced ability to update lower-level beliefs (e.g., predictions about performance), even when reward contingencies change, indicating the influence of rigid metacognitive priors (Schneider et al., 2024). To validate the proposed model, we suggest a series of experimental paradigms that isolate the various mechanisms contributing to depressive belief formation. Biases toward negative information could be explored with tasks exposing participants to environments containing different proportions of aversive and neutral explicit and implicit stimuli. Moreover, integrating hierarchical belief networks into our experimental designs necessitates the development of tasks that assess the impact of mood on both high-level metacognitive beliefs (e.g., beliefs about success or failure) and lower-level predictions (e.g., expectations regarding task performance).

The Hierarchical Gaussian Filter, a computational model used in Bayesian inference, has been used precisely to model the dynamics of beliefs in hierarchical systems, describing how beliefs are constructed and updated across different levels, ranging from low-level sensory perceptions to high-level cognitive representations (Henco et al., 2020; Kreis et al., 2023). However, it faced challenges in providing reliable measurements of belief updating at all levels simultaneously, with the limitation of a high correlation between hierarchical parameters, which complicates the interpretation of specific levels (Mathys et al., 2014). To address this limitation, we propose modifications to existing tasks that would allow for more reliable measurements of belief updating across different hierarchical levels: first, by incorporating multimodal calibration that combines behavioral measures (prediction tasks) with physiological measures (e.g., eye tracking); and second, by modularizing hierarchical levels through experimental blocks that isolate each belief level before their gradual integration.

Besides, integrating mood into the Hierarchical Gaussian Filter framework provides a promising approach to studying the dynamic interplay between affective states and belief updating processes. First, mood could serve as a modulating factor that affects sensitivity to prediction errors at each level, influencing update equation parameters such as sensitivity to phasic changes and precision weights based on mood states. For example, negative prediction errors might be weighted more heavily, reinforcing pessimistic beliefs, while positive mood states could favor more optimistic updates. Mood could also affect the temporal dynamics of belief revision by adjusting the learning rate, with negative mood states potentially slowing belief updating and contributing to cognitive rigidity. Additionally, mood could be incorporated as an additional hierarchical layer modulating volatility estimates (Clark et al., 2018), capturing how emotional states influence belief updating processes.

Finally, biases related to cognitive feedback loops could be explored using tasks that exclude sensory feedback (i.e., in the absence of external evidence), allowing for the creation of evidence-free tasks to quantify the persistence of meta-beliefs without sensory input (in contrast to classical paradigms). For instance, tasks might ask participants to rate the likelihood of experiencing negative events in the future, even when no new information is provided (Gagne et al., 2022). To refine this task, we propose introducing a systematic manipulation of emotional valence at each hierarchical level, along with non-Markovian transitions between levels. These transitions enable the modeling of belief updating processes that rely not only on the most recent observation but also on a broader history of prior states. By incorporating non-Markovian transitions, we reduce parametric confounds and more effectively disentangle the effects of hierarchical emotional valence manipulations from biases in belief updating. This approach enhances the task’s ecological validity, provides a more precise computational account of mood-congruent belief formation, and yields testable predictions regarding the interaction between belief updating and mood.

### Targeting belief updating bias in depression

A better understanding of the computational mechanisms of belief updating could lead to improved therapeutic strategies for MDD. While current interventions often target the content of negative beliefs (e.g., through cognitive restructuring in cognitive behavioral therapy), cognitive theories of belief formation emphasizes the importance of recalibrating the hierarchical inference processes that generate and sustain these beliefs. Considering that negative beliefs can often be quite entrenched and resistant to change even in the face of positive experiences that disconfirm these beliefs, it is crucial to develop alternative strategies to modulate belief updating bias (Kube et al., 2017).

For instance, psychotherapies that address these underlying processes, such as Metacognitive Therapy (MCT), assist individuals in challenging and modifying the way they update their beliefs, rather than simply disputing the content of negative thoughts (Callesen et al., 2020; Schneider et al., 2024). These therapies focus on how individuals engage with their thoughts and the metacognitive processes that govern belief revision, promoting more adaptive methods of belief updating. Additionally, cognitive theory of belief formation predicts that enhancing sensitivity to sensory prediction errors—through tasks that train individuals to detect and integrate novel sensory inputs—could directly address impaired precision-weighting (Moutoussis et al., 2018). These insights emphasize the need to focus on belief updating processes in psychotherapy to gain a deeper understanding of therapeutic changes, moving beyond the approaches suggested by reward-deficit theories, which mainly aim to increase engagement with positive stimuli or rewards (Admon & Pizzagalli, 2015).

Pharmacological treatments may also play a role in modulating precision-weighting by altering glutamatergic, dopaminergic, and serotonergic signaling, which is thought to influence the balance between prior beliefs and sensory evidence (Bottemanne et al., 2023). Studies have shown that monoaminergic antidepressants, such as SSRIs, modify the perception of positive signals by increasing sensitivity to positive emotional information (Godlewska & Harmer, 2020). By improving the recognition and processing of positive stimuli, these antidepressants may help to counteract the negative biases characteristic of depression (Pringle et al., 2011). This effect could influence the mechanisms of belief updating, potentially facilitating a more adaptive revision of beliefs by incorporating positive experiences and reducing the dominance of negative, maladaptive thought patterns (Harmer & Browning, 2020).

Conversely, a study has shown that ketamine, an NMDA receptor antagonist transiently promoting glutamatergic transmission, directly alters the belief updating bias by reducing the updating of negative information several hours after administration (Bottemanne et al., 2022b). This effect suggests that ketamine may specifically dampen the processing of negative signals, potentially reshaping belief revision by limiting the incorporation of negative feedback into cognitive frameworks (Bottemanne et al., 2023). Furthermore, the early modification of the belief updating bias predicted the antidepressant efficacy of the treatment after 1 week (Bottemanne et al., 2022b). This suggests that changes in how individuals process and update beliefs, particularly in response to negative information, could serve as an early marker for the therapeutic effectiveness of the treatment. Based on these findings, combining pharmacological interventions with therapies aimed at modulating hierarchical inference could create a synergistic effect, enhancing treatment outcomes by addressing both the neurobiological and cognitive aspects of belief updating.

## Conclusions

We present a mechanistic theory of belief updating in MDD at the individual level, showing how it occurs across three key dimensions. First, we show how depressive beliefs are formed in environments where negative stimuli are weighted more heavily. Second, we clarify how depressed individuals hold rigid negative metacognitive priors that inhibit belief updating. Third, we explain how negative beliefs can be generated internally through repetitive, self-focused thought patterns. Future research should explore how these factors interact and further investigate their role in mood disorders.

## Data Availability

Not applicable.
